# The Relationship between Number of Supernumerary Blastocysts Cryopreserved and Probability of a Live Birth Outcome after Single Fresh Blastocyst Transfer: Analysis of over 10 Thousand Cycles

**DOI:** 10.3390/jcm12134172

**Published:** 2023-06-21

**Authors:** Yusuf Beebeejaun, Timothy Copeland, Lukasz Polanski, Tarek El Toukhy

**Affiliations:** 1 Department of Women’s Health, Faculty of Life Sciences and Medicine, King’s College London, London WC2R 2LS, UK; tarek.el-toukhy@gstt.nhs.uk; 2 Assisted Conception Unit, Guy’s and St Thomas’ Hospital, London SE1 9RT, UK; lukasz.polanski@nhs.net; 3 Department of Health Policy & Management, Fielding School of Public Health, University of California, Los Angeles, CA 90095, USA

**Keywords:** IVF, blastocyst transfer, cryopreservation, live birth, age

## Abstract

The ability to predict the likelihood of a live birth after single fresh embryo transfer is an important part of fertility treatment. While past studies have examined the likelihood of live birth based on the number of oocytes retrieved and cleavage-stage embryos available, the odds of a live birth based on the number of supernumerary blastocysts cryopreserved following a fresh embryo transfer has not been rigorously studied. We performed a retrospective analysis, stratified by age, on patients undergoing their first fresh autologous single day 5 blastocyst transfer to assess relationship between the likelihood of a live birth and number of supernumerary blastocysts cryopreserved. In patients aged <35 years and 35–39 years old, the likelihood of a live birth increased linearly between 1 and 6 supplementary blastocysts and non-linearly if 10 or more blastocysts were cryopreserved. When aged 40 years and above, the likelihood of a live birth increased linearly up to 4 cryopreserved blastocysts and then non-linearly if 10 or more blastocysts were cryopreserved. The present study demonstrated a non-linear relationship between the number of supernumerary blastocysts cryopreserved and the likelihood of a live birth after single blastocyst transfer in the first autologous fresh IVF/ICSI cycle across different age groups.

## 1. Introduction

The ability to predict the likelihood of a live birth after single fresh embryo transfer is important for treatment planning and managing patient expectation, particularly in the first in vitro fertilisation (IVF) cycle. Cryopreservation of supernumerary embryos is often regarded as an important prognostic variable and a surrogate marker of IVF success. It increases the cumulative pregnancy rate from a single IVF stimulation [1] permits the transfer of fewer embryos per attempt [2], and decreases the costs associated with multiple IVF stimulation cycles.

The overall reduction in the mean number of embryos transferred over the last decade, in conjunction with improvement in extended embryo culture and cryopreservation techniques, have led to an increase in the availability of surplus embryos for cryopreservation [3].

Previous large studies have examined the association between the number of oocytes retrieved and cleavage-stage embryos available, and the odds of a live birth following a fresh embryo transfer [4,5,6,7]. However, the number of oocytes retrieved does not always predict the number of blastocysts eventually available for transfer and freezing, and therefore could be a less reliable marker of embryonic implantation potential and IVF outcome. The number of supernumerary blastocysts suitable for freezing is therefore perceived as a stronger predictor of cycle outcome. Whilst previous studies have described the association between the occurrence of a live birth and embryo availability [8,9], the effect of patient’s age on this association, which is often the biggest confounder of success within an IVF cycle, has not been rigorously studied.

The aim of this study was to examine the relationship between the number of supernumerary blastocysts cryopreserved and the likelihood of achieving a live birth following elective fresh autologous single blastocyst transfer in patients having their first IVF cycle, stratified according to patient age.

## 2. Materials and Methods

### 2.1. Study Design and Participants

Anonymised data on IVF cycles performed at the Assisted Conception Unit, at Guy’s and St Thomas’ Hospital in London, United Kingdom, were collected between July 2006 and June 2018 and retrospectively analysed. This cohort study included records of patients who had their first IVF and intra-cytoplasmic sperm injection (ICSI) cycle culminating in a fresh autologous single blastocyst transfer on day 5 after oocyte fertilisation. All patients included within this study who had at least one supernumerary blastocyst cryopreserved underwent elective single blastocyst transfer.

To minimise potential confounders, strict inclusion and exclusion criteria were utilised. Inclusion criteria included women aged between 20 and 45 years old undergoing their first fresh stimulation cycle of IVF or ICSI. Cycles involving pre-implantation genetic testing (PGT), oocyte donation, transfer of cleavage-stage or more than one fresh embryo or total embryo freezing due to increased risk of ovarian hyperstimulation syndrome were excluded.

Patient demographics such as female age, body mass index (BMI), previous fertility treatment, previous live birth, period of treatment, and cycle characteristics, including total dose of gonadotrophins administered, method of oocyte fertilisation (IVF vs. ICSI), number of oocytes retrieved and normally fertilised, and number of supernumerary blastocysts cryopreserved in the fresh cycle were analysed.

Since age is known to influence the likelihood of achieving a live birth after fresh embryo transfer [10], we stratified patients based on their age into three groups (younger than 35 years, 35–39 years, and 40 years and older). The time period of July 2006 until June 2018 was selected as no major changes in clinical protocols, culture conditions, or laboratory equipment were made during this time period.

### 2.2. Ovarian Stimulation and Oocyte Retrieval

Follicle stimulating hormone (FSH) injections were started at a dose of 150–450 IU daily for multi-follicular ovarian stimulation in either the mid-luteal long down regulation or the short gonadotropin-releasing hormone (GnRH) antagonist protocol as described previously [11,12]. The choice of the controlled ovarian stimulation protocol and daily dose of the follicle stimulating hormone (FSH) injections was based on female age, basal follicle stimulating hormone (FSH) level, AFC and anti-mullerian hormone (AMH) level as described elsewhere [12,13].

Ovarian response was monitored by transvaginal ultrasound scanning and, where required, serum estradiol level measurements were taken and oocyte maturation was induced using either 10,000 IU of urinary-derived HCG (Pregnyl, Merck-Serono, Halle, Germany) or 250 µg of recombinant HCG (Ovitrelle, Merck-Serono, Darmstadt, Germany) when at least three 18 mm follicles were seen on ultrasound scan.

Transvaginal ultrasound-guided retrieval of cumulus–oocyte complexes was performed using GE Logic V1 portable scanning system (GE Healthcare Ultrasound, Milwaukee, WI, USA) 36 h after the HCG injection.

### 2.3. Embryo Assessment

Standard IVF or ICSI was used for oocyte fertilisation as clinically indicated and embryos were cultured until day 5 after fertilisation if at least 2 embryos were deemed to be of good quality on day 3 after fertilisation as previously described [14]. On day 5 after fertilisation, fresh blastocyst-stage embryos were assigned grades according to strict morphological criteria [15,16], which were not changed during the study period. According to our laboratory practice and unpublished audit data, an embryo was considered suitable for transfer or freezing if it had reached the expanded blastocyst stage with prominent and compact inner cell mass and many identical trophectoderm cells forming a continuous layer [blastocyst grade 3CC or higher] and showed no signs of degeneration [15,16].

All patients included in the study had single embryo transfer in accordance with national regulations [17]. All embryo transfers were performed on day 5 after fertilisation under transabdominal ultrasound guidance using GE Voluson 730 system (GE Healthcare Ultrasound). Supernumerary blastocysts of grade 3CC or higher that showed no signs of degeneration were cryopreserved on day 5 or 6 after fertilisation. A standard slow freezing protocol, employing 1,2-propanediol (PROH) and sucrose as cryoprotectants [18,19], or vitrification using Cryolock device (Biotech Inc., Alpharetta, GA, USA) and Vitrolife vitrification medium (Vitrolife Ltd., Warwick, UK) was used for blastocyst cryopreservation. The luteal phase was supported with 400 mg progesterone pessaries (Cyclogest, Actavis UK Ltd., Barnstaple, UK) twice daily commencing on the day of oocyte retrieval until 8 weeks’ gestation.

### 2.4. Outcome Measure

The outcomes of each treatment cycle included pregnancy, clinical pregnancy, and live birth. Pregnancy was confirmed by a positive urine hCG test 11 days after blastocyst transfer. A clinical pregnancy was defined as the observation of a gestational sac on ultrasound scanning 5 weeks after embryo transfer. Live birth was defined as the delivery of a live born baby after 23 completed weeks of gestation. For this study, live birth was the primary outcome of interest.

### 2.5. Statistical Analysis

The study data were presented as median ± interquartile range (IQR) or percentage. Statistical analysis was performed using STATA version 16 [20]. The probability of live birth between patients with supernumerary blastocysts cryopreserved and those without was performed through a chi-square analysis. Continuous parameters were compared using Wilcoxon rank-sum tests. A multivariate logistic regression analysis was used to examine the association between live birth and whether or not supernumerary blastocysts were available for cryopreservation after controlling for important confounders, including female age, protocol and type of gonadotrophins used for ovarian stimulation, total dose of gonadotrophins administered, and number of oocytes retrieved.

To assess the association between the number of supernumerary blastocysts cryopreserved and probability of a live birth outcome, fractional logistic regressions [21] were modelled using a cubic polynomial transformation of the number of supernumerary blastocysts cryopreserved, stratified by female age group. With the exception of the histogram of number of blastocysts, the x-axes and row numbers in figures and tables have been truncated at 15 for legibility, since a small number of cycles (*n* = 12) had over 15 (16–30) supernumerary blastocysts cryopreserved. The Shapiro–Wilk test was performed to assess and confirm the normality of continuous variables.

### 2.6. Ethics

Ethical approval was received for this study from the local research ethics committee (ref: 01121432/2019). Our study involved neither therapeutic intervention nor change of our routine IVF protocols or data collection. Each couple gave written informed consent for the use of their data anonymously for audit and research purposes upon enrolment into our IVF programme and before starting an IVF cycle in accordance with the UK Human Fertilisation and Embryology Authority (HFEA) regulations.

## 3. Results

A total of 10,015 fresh autologous non-PGT IVF/ICSI cycles performed in our centre between July 2006 and June 2018 were eligible for inclusion in the study. The mean female age of couples included in the study cohort was 35.1 ± 4.4 years (range 18–45) and was normally distributed throughout the dataset.

**Figure 1:**
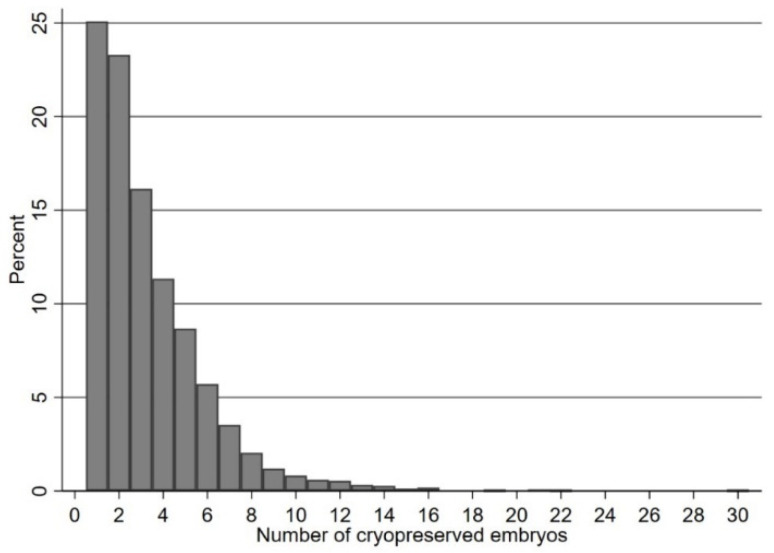
Distribution of the number of cryopreserved embryos among cycles included in this study.

The mean daily dose of FSH used for ovarian stimulation was 283.8 ± 54.5 IU (range 112.5–450), and the mean duration of ovarian stimulation was 11.2 ± 2.1 days. In 64% of cycles, ICSI was the method used for oocyte fertilisation. The mean number of normally fertilised oocytes was 12.1 ± 7.0. Of the 10,015 first fresh IVF/ICSI cycles included in the study, 5992 (60%) had a single blastocyst transfer with no supernumerary blastocysts available for cryopreservation, whilst 4023 cycles (40%) had between 1 and 30 (mean 3.3 ± 2.5) supernumerary blastocysts cryopreserved. Figure 1 depicts the frequency distribution of the number of cryopreserved blastocysts in those 4023 cycles.

The overall clinical pregnancy and live birth rates in the study were 36% and 30%, respectively. Cycles in which one or more supernumerary blastocysts were cryopreserved had a significantly higher live birth rate compared to those without blastocyst cryopreservation (38% vs. 24%, *p* < 0.0001, Table 1). After adjusting for important confounders, the likelihood of achieving a live birth following a single blastocyst transfer in cycles where one or more supernumerary blastocysts were cryopreserved was significantly higher compared to those without blastocyst cryopreservation (adjusted odds ratio (aOR) 1.76, 95% CI: 1.61–1.92, *p* < 0.0001).

### 3.1. Association of Live Birth Rate with Number of Supernumerary Blastocysts Cryopreserved

**Figure 2:**
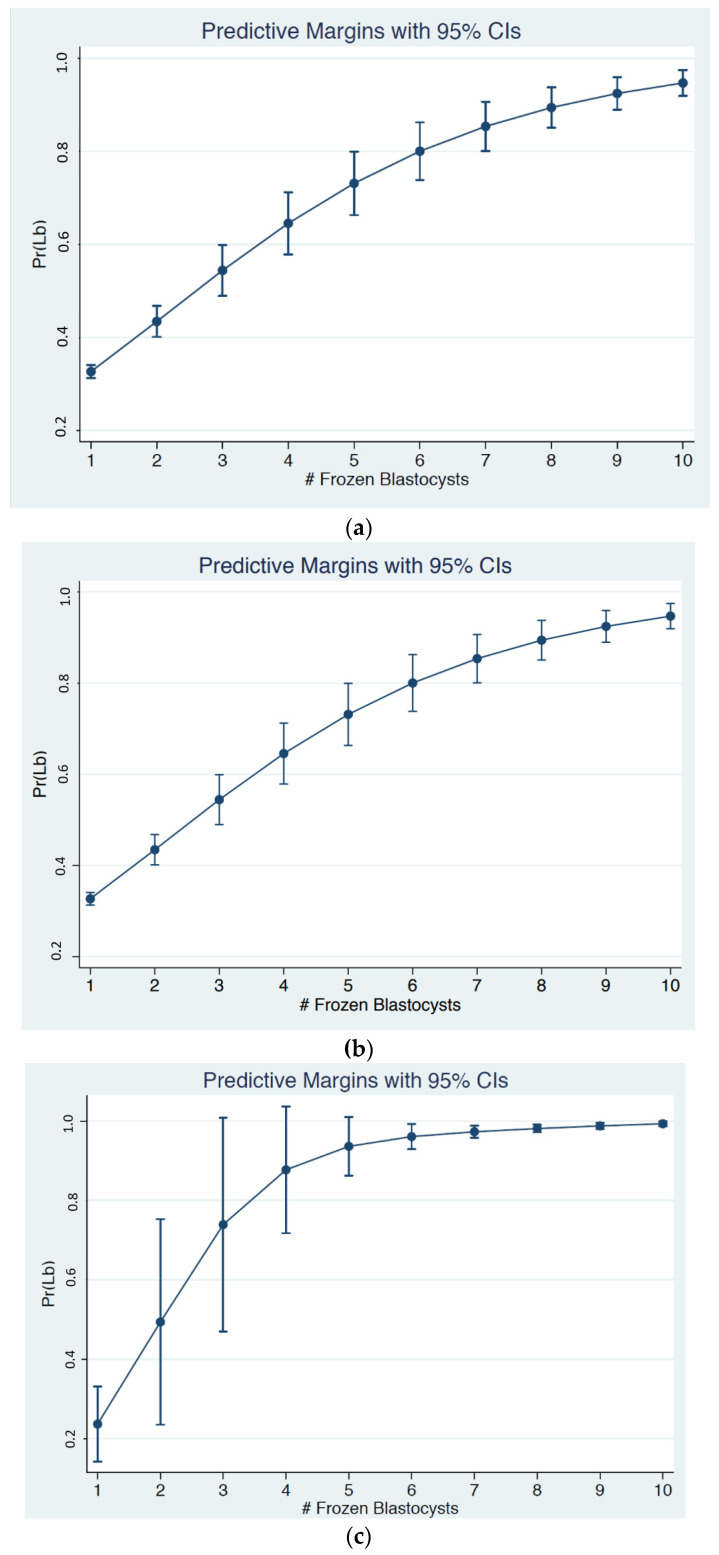
Predicted odds of a live birth by number of cryopreserved embryos, stratified by age: (**a**) in women aged less than 35 years, (**b**) in women aged between 35 and 30 years, (**c**) in women aged more than 40 years.

We further analysed the relationship between the number of supernumerary blastocysts cryopreserved and the odds of a live birth following fresh single blastocyst transfer using a cubed polynomial regression model. To aid clinical relevance, we stratified the regression model according to three female age groups (<35, 35–39, and ≥40 years). Figure 2 shows the probability of a live birth in relation to the number of blastocysts cryopreserved within each age group. The confidence intervals of the predicted live birth rates began to converge at 10 or more cryopreserved blastocysts. Given the statistical convergence and the relatively small sample size above 10 cryopreserved embryos, these intervals are not displayed. All three age groups had statistically significant cubed number of blastocysts terms, indicating departure from a strictly linear relationship, and favouring a curvi-linear association instead (Figure 2).

### 3.2. Probability of Live Birth When Female Age Is below 35 Years

For those aged below 35 years, the likelihood of having a live birth increased for each additional blastocyst cryopreserved in a linear progression from 0.33 (95% CI 0.31–0.34) if one supernumerary blastocyst was cryopreserved to 0.80 (95% CI 0.74–0.86; *p* < 0.0001) if six blastocysts were cryopreserved, with an average increase in the live birth rate of 7.8% with each additional blastocyst cryopreserved up to six. The likelihood of having a live birth then increased non-linearly, to 0.95 (95% CI 0.92–0.97; *p* < 0.0001) if 10 or more blastocysts were cryopreserved, with an average increase in the live birth rate of 3.8% with each additional blastocyst cryopreserved after 6 up to 10 or more.

### 3.3. Probability of Live Birth When Female Age Is between 35 and 39 Years

For those aged between 35–39 years, the likelihood of having a live birth increased for each additional blastocyst cryopreserved in a linear progression from 0.30 (95% CI 0.28–0.32) if one supernumerary blastocyst was cryopreserved to 0.82 (95% CI 0.73–0.91; *p* < 0.0001) if six blastocysts were cryopreserved, with an average increase in the live birth rate of 8.6% with each additional blastocyst cryopreserved up to six. The likelihood of having a live birth then increased non-linearly to 0.96 (95% CI 0.93–0.99; *p* < 0.0001) if 10 or more blastocysts were cryopreserved, with an average increase in the live birth rate of 3.5% with each additional blastocyst cryopreserved after 6 up to 10 or more.

### 3.4. Probability of Live Birth When Female Age Is Aged 40 Years and Above

The likelihood of having a live birth increased for each additional blastocyst cryopreserved in a linear progression from 0.26 (95% CI 0.19–0.32) if one supernumerary blastocyst was cryopreserved to 0.83 (95% CI 0.68–0.97; *p* < 0.0001) if four blastocysts were cryopreserved, with an average increase in the live birth rate of 14.3% with each additional blastocyst cryopreserved up to four. The likelihood of having a live birth then increased non-linearly to 0.99 (95% CI 0.98–0.99; *p* < 0.0001) if 10 or more blastocysts were cryopreserved, with an average increase in the live birth rate of 2.7% with each additional blastocyst cryopreserved after 4 up to 10 or more.

## 4. Discussion

To date, this is the largest study evaluating the relationship between the number of supernumerary blastocysts cryopreserved and the probability of a live birth after day 5 autologous non-PGT single fresh blastocyst transfer in women having their first IVF/ICSI cycle, and the impact of age on this relationship, using data from one IVF centre. Our results indicate that achieving blastocyst cryopreservation is associated with a significantly higher live birth rate and that there is a curvi-linear positive correlation between the number of supernumerary blastocysts cryopreserved and the probability of a live birth until 10 or more blastocysts are cryopreserved. This correlation remained valid at different age groups, suggesting that a cohort effect of human embryos exists in IVF/ICSI cycles at various age groups, including those aged 40 years and older [22]. It also indicates that ovarian stimulation may not have a detrimental impact on either oocyte/embryo quality or endometrial receptivity, lending support to similar observations in recent studies [23,24,25]

The results of the present study are consistent with previous studies reporting a positive correlation between achieving embryo cryopreservation and clinical IVF outcomes, after both cleavage-stage embryo [26] and blastocyst transfer [27]. Our results also corroborate those of a much smaller study [8], which included 655 fresh autologous single blastocyst transfers in good prognosis patients and reported a positive correlation between the number of supernumerary blastocysts cryopreserved and the odds of a live birth after the fresh blastocyst transfer. However, this study did not limit the analysis to patients having their first IVF/ICSI cycle; it included patients who had day 6 blastocyst transfer and did not evaluate the results according to specific age groups [8].

In addition, our results are consistent with the study of Papanikolou et al. (2019) [9], which included 244 women who had blastocyst-stage embryo transfer and reported that the highest live birth rate after the first transfer was achieved in women who had the greatest number of blastocysts cryopreserved (seven or more blastocysts) compared to those who had fewer blastocysts cryopreserved.

The results of the present study contrast with those of a recent report [28] which suggested that the likelihood of a live birth after single blastocyst transfer increased with the increase in the total number of blastocysts available up to five blastocysts, then decreased for every additional blastocyst available thereafter in patients up to age 35 years, but not in those aged 36–40 years. Unlike our study, the study of Smeltzer and colleagues analysed the number of all, rather than supernumerary, blastocysts generated in an IVF/ICSI cycle and excluded patients above the age of 40 years. Furthermore, the drop in live birth rate after five supernumerary embryos generated is not consistent with recent reports demonstrating an increase in the cumulative live birth rate with the increased availability of oocytes and embryos in the fresh cycle [5,23,29,30,31,32]. In addition, the dataset analysed in the study of Smeltzer and colleagues (2019) was based on a national database registry, reflecting different clinical and laboratory IVF practices, thus potentially introducing a degree of performance and reporting bias. Furthermore, the study of Smeltzer and colleagues suggested that patients aged 35 years or younger who have a total of more than five blastocysts available in a given IVF/ICSI should undergo PGT for aneuploidy (PGT-A) to better select the blastocyst suitable for transfer, whereas a recent randomised trial [33] demonstrated that PGT-A did not improve the overall pregnancy outcomes in women who have two or more blastocysts suitable for biopsy, particularly in those aged 35 years or younger.

Our study results are clinically relevant as they can be used to improve counselling of couples at the time of their first fresh embryo transfer by providing a realistic perspective of the probability of delivering a baby after single blastocyst transfer based on female age and the number of supernumerary blastocysts cryopreserved. Understanding the association between the probability of a live birth and the number of supernumerary blastocysts cryopreserved could support a practice of single fresh blastocyst transfer when multiple supernumerary blastocysts are cryopreserved irrespective of patient age, and thus help to reduce the temptation to transfer more than one blastocyst, thereby reducing the overall risk of multiple births.

The strengths of the present study include the analysis of a large number of IVF/ICSI cycles performed without significant changes in clinical or laboratory protocols. In addition, by only including patients undergoing single blastocyst transfer in their first IVF/ICSI cycle and excluding patients undergoing cleavage-stage embryo transfer, PGT, or receiving donated oocytes, we analysed a relatively homogenous cohort of patients, thereby accounting for potential treatment modality confounders, as well as removing the influence of previous IVF cycles on the utilisation of embryos available in a second or subsequent cycle. Furthermore, we stratified our results by age group to guide clinical practice more precisely and enable clinicians to provide better counselling to patients embarking on their first fresh embryo transfer.

However, one distinct limitation of our study was the lack of data on clinical pregnancy loss. We also did not have data reporting on sperm characteristics or the euploid status of the transferred embryos or that of the cryopreserved embryos. While the use of pre-implantation genetic testing for aneuploidy (PGT-A) has been reported to potentially improve live birth rates when used as a tool for embryo selection, embryo selection was carried out solely through morphological evaluation. This study can therefore not be interpreted in the context of having completely euploid embryos cryopreserved. Another limitation of the study is the fact that we could not stratify the results into smaller age brackets as this would have had an impact on the statistical significance of the results produced. It also included results from women undergoing both either the mid-luteal long down regulation or the short gonadotropin-releasing hormone (GnRH) antagonist protocol.

In conclusion, the present study results demonstrate that the number of supernumerary blastocysts cryopreserved in the first autologous fresh IVF/ICSI cycle can be a key factor in determining the probability of achieving a live birth after single blastocyst transfer across different age groups. This information can be helpful in patient counselling and promoting single embryo transfer to reduce the risk of multiple births. This study shows that the presence of additional good-quality supernumerary embryos can be used as a reference to determine the likelihood of success following an embryo transfer. Additionally, this study supports a single embryo transfer which is based on embryo quality, maternal age, and the presence or absence of surplus quality embryos.

## Figures and Tables

**Table 1:** Baseline characteristics and IVF cycle outcome in those having supernumerary cryopreserved embryos vs. those who did not. Values are provided as median (IQR) or as percentage.

Factor	No Blastocysts Cryopreserved	Supernumerary Blastocysts Cryopreserved	*p*-Value
Number	5992	4023	
Age, median (IQR)	36.0 (33.0, 39.0)	34.0 (31.5, 37.0)	<0.001
Age Group			
<35	2256 (38%)	2119 (53%)	<0.001
35–39	2970 (49%)	1728 (43%)	
≥40	766 (13%)	176 (4%)	
Baseline FSH (IU) Level, median (IQR)	6.3 (5.3, 7.7)	6.3 (5.3, 7.6)	0.15
Type of Gonadotrophins used			
Recombinant	5347 (89.2%)	3688 (91.7%)	<0.001
Urinary-derived	645 (10.8%)	335 (8.3%)	
Daily Dose of FSH, median (IQR)	277 (150.0, 300.0)	225.0 (150.0, 300.0)	<0.001
Duration of Ovarian Stimulation, median (IQR)	11.0 (10.0, 12.0)	11.0 (10.0, 12.0)	0.33
Total Dose of FSH, median (IQR)	2700 (1800, 3600)	2250 (1650, 3150)	<0.001
Number of Oocyctes Retrieved, median (IQR)	9.0 (6.0, 14.0)	14.0 (10.0, 18.0)	<0.001
Method of Oocyte Fertilisation			
IVF	2105 (35%)	1461 (36%)	
ICSI	3887 (65%)	2562 (64%)	
No. of Normally-Fertilised Oocytes, median (IQR)	5.0 (3.0, 8.0)	9.0 (6.0, 12.0)	<0.001
Clinical Pregnancy	1568 (28%)	1612 (44%)	<0.001
Live Birth	1436 (24%)	1518 (38%)	<0.001

## Data Availability

The data presented in this study are available on request from the corresponding author. The data are not publicly available due to the nature of the study and for patient confidentiality.
